# Design and conduct of Caudwell Xtreme Everest: an observational cohort study of variation in human adaptation to progressive environmental hypoxia

**DOI:** 10.1186/1471-2288-10-98

**Published:** 2010-10-21

**Authors:** Denny ZH Levett, Daniel S Martin, Mark H Wilson, Kay Mitchell, Sundeep Dhillon, Fabio Rigat, Hugh E Montgomery, Monty G Mythen, Michael PW Grocott

**Affiliations:** 1 Centre for Altitude Space and Extreme Environment Medicine, UCL Institute of Human Health and Performance, First Floor, Charterhouse Building, UCL Archway Campus, Highgate Hill, London, N19 5LW, UK; 2 Centre for Altitude Space and Extreme Environment Medicine, Portex Applied Human Physiology, UCL Institute of Child Health, 30 Guilford Street, London WC1N 1EH, UK; 3 Department of Statistics and Centre for Analytical Science, University of Warwick, Gibbet Hill road, CV4 7AL Coventry, UK; 4 Smiths Medical Professor of Anaesthesia and Critical Care, Portex Unit, UCL Institute of Child Health, 30 Guilford Street, London WC1N 1EH, UK

## Abstract

**Background:**

The physiological responses to hypoxaemia and cellular hypoxia are poorly understood, and inter-individual differences in performance at altitude and outcome in critical illness remain unexplained. We propose a model for exploring adaptation to hypoxia in the critically ill: the study of healthy humans, progressively exposed to environmental hypobaric hypoxia (EHH). The aim of this study was to describe the spectrum of adaptive responses in humans exposed to graded EHH and identify factors (physiological and genetic) associated with inter-individual variation in these responses.

**Methods:**

**Design**

Observational cohort study of progressive incremental exposure to EHH.

**Setting**

University human physiology laboratory in London, UK (75 m) and 7 field laboratories in Nepal at 1300 m, 3500 m, 4250 m, 5300 m, 6400 m, 7950 m and 8400 m.

**Participants**

198 healthy volunteers and 24 investigators trekking to Everest Base Camp (EBC) (5300 m). A subgroup of 14 investigators studied at altitudes up to 8400 m on Everest.

**Main outcome measures**

Exercise capacity, exercise efficiency and economy, brain and muscle Near Infrared Spectroscopy, plasma biomarkers (including markers of inflammation), allele frequencies of known or suspected hypoxia responsive genes, spirometry, neurocognitive testing, retinal imaging, pupilometry. In nested subgroups: microcirculatory imaging, muscle biopsies with proteomic and transcriptomic tissue analysis, continuous cardiac output measurement, arterial blood gas measurement, trans-cranial Doppler, gastrointestinal tonometry, thromboelastography and ocular saccadometry.

**Results:**

Of 198 healthy volunteers leaving Kathmandu, 190 reached EBC (5300 m). All 24 investigators reached EBC. The completion rate for planned testing was more than 99% in the investigator group and more than 95% in the trekkers. Unique measurements were safely performed at extreme altitude, including the highest (altitude) field measurements of exercise capacity, cerebral blood flow velocity and microvascular blood flow at 7950 m and arterial blood gas measurement at 8400 m.

**Conclusions:**

This study demonstrates the feasibility and safety of conducting a large healthy volunteer cohort study of human adaptation to hypoxia in this difficult environment. Systematic measurements of a large set of variables were achieved in 222 subjects and at altitudes up to 8400 m. The resulting dataset is a unique resource for the study of genotype:phenotype interactions in relation to hypoxic adaptation.

## Background

A fall in cellular oxygen use occurs in diverse disease states: oxygen uptake may be reduced (pulmonary disease), its mass transport diminished (cardiac contractile failure or anaemia), or its local delivery (microvascular disease) or cellular use (cellular dysoxia) impaired. In the critically ill patient many or all of these factors may exist simultaneously [1]. The (patho)physiological responses to hypoxaemia and cellular hypoxia are far from understood, and inter-individual differences in performance at altitude and outcome in critical illness remain unexplained. This lack of understanding stems partly from difficulties in dissecting the pathways of hypoxic adaptation in pathophysiological states. In critical illness, patient demographics, presenting conditions, co-morbidities and therapies vary greatly, and multiple biological characteristics are disturbed. Defining the specific effects of hypoxia, a single component in a multi-factorial disease state, is therefore difficult. Clarifying whether a change in a measured variable is the cause of the hypoxia, or a response to it, is harder still.

Currently available models of critical illness are recognised to have substantial limitations [1-4]. We therefore propose an alternative model for exploring adaptation to hypoxia in the critically ill: the study of healthy humans, progressively exposed to environmental hypobaric hypoxia in a controlled manner, during an ascent to high altitude [1,5].

Barometric pressure falls progressively with increasing altitude whilst the fractional inspired concentration of oxygen remains constant resulting in a fall in the inspired partial pressure of oxygen (P_I_O_2_). At Everest Base Camp (5300 metres), the ambient (atmospheric) partial pressure of oxygen (P_AT_O_2_) is approximately half that at sea level, and on Everest's summit (8848 m) it is one third of the sea level value.

Previous field studies at high altitude have provided data on human adaptation to hypoxia in small groups (n = 12-36) of highly selected subjects [6-9]. Whilst larger prospective studies have been conducted by groups such as Medical Expeditions (MEDEX), ascent rates varied between subjects and hypoxic exposure was not standardized [10-12]. Studies in large groups with tightly controlled ascent profiles (ie identical hypoxic exposure) have not previously been conducted. Furthermore, developments in molecular genetics, along with novel technologies for physiological measurement, allow new questions to be addressed and genotype:phenotype relationships to be explored [13]. This approach may provide useful data to inform our understanding of adaptation in situations where hypoxaemia/cellular hypoxia is a fundamental problem.

The aim of Caudwell Xtreme Everest (CXE) [14] was to study, comprehensively and prospectively, a large cohort of healthy humans exposed to progressive, sustained environmental hypobaric hypoxia. The results will be used to drive a translational research agenda, with the aim of developing novel diagnostic and therapeutic interventions for patients. Core hypotheses are as follows: (1) Mechanisms distinct from those related to global oxygen transport will in part explain inter-individual differences in adaptation (functional capacity, organ specific adaptation, absence of altitude illness) at high altitude, (2) genotype differences will explain a substantial proportion of intra-individual variation in environmentally induced phenotypes (gene-environment interactions). Possible mechanisms underlying hypothesis one include alterations in metabolic efficiency and changes in microcirculatory function. Subsidiary aims include exploring the interaction between hypoxia and inflammation, identifying biomarkers associated with beneficial and adverse hypoxic adaptation, and characterising the physiological state of well-adapted individuals close to the limit of hypoxia tolerance above 7500 metres. This paper describes the design and conduct of the CXE study. We report the pattern of hypoxic exposure, characteristics of the subject groups, and reasons for subject drop-outs. We also discuss the strengths and limitations of the proposed model.

## Methods

CXE study design, risk management plan and individual protocols were approved by the University College London Research Ethics Committee (in accordance with the declaration of Helsinki). Verbal and written informed consent was obtained from all subjects. The study took place between January and June 2007. The study was initiated, designed and conducted by the UCL Centre for Altitude Space and Extreme Environment Medicine (CASE). All funding was unrestricted. All authors read and approved this manuscript.

### Study Participants

Eligible adults (candidate subjects) were aged over 18 years (no upper age limit) - the females not being pregnant - and were required to pass two separate health-screening stages. Candidate subjects with diabetes mellitus, significant cardiac or significant respiratory disease were excluded. Diabetes mellitus was defined as a requirement for control of blood glucose either by diet, oral hypoglycaemics or insulin therapy. Significant respiratory disease was defined as disease that could deteriorate at altitude and render the subject at risk during the trek to Everest Base Camp. For example well-controlled, mild asthma was not an exclusion criterion; severe chronic obstructive airways disease was. Significant cardiac disease was defined as disease that could deteriorate at altitude and render the subject at risk during the trek. For example ischaemic heart disease with angina and symptomatic heart failure were excluded; controlled hypertension was not. Doctors qualified in mountain medicine and working for the company responsible for travel arrangements to Everest Base Camp performed the initial screening. They reviewed the medical screening forms that were completed by all subjects and determined fitness for travel to altitude. The CXE medical officer (an experienced expedition doctor, DL) independently confirmed fitness to travel to altitude, as well as fitness to participate in the research studies. In cases where further medical information was required, DL contacted the candidate subjects directly and, where appropriate and with consent, their medical practitioners.

Additional exclusion criteria for cardiopulmonary exercise testing (CPET) were based on the American Thoracic Society/American College of Chest Physicians guidelines for clinical exercise testing [15]. All subjects with an absolute or relative contraindication, as defined by these guidelines, were excluded from this protocol. In addition any individual diagnosed by the expedition medical team with altitude illness was excluded from CPET whilst symptoms were present and treated appropriately. Subjects' resting physiological variables (oxygen saturation, pulse, blood pressure and electrocardiogram) were monitored prior to the commencement of exercise testing by investigators. Specific altitude dependent symptom and physiological criteria were used to trigger referral to the expedition medical team for assessment and consideration of exclusion from CPET testing at that laboratory (Table 1).

**Table 1:** Criteria for exclusion from exercise testing at field laboratories and for stopping tests.

Exclusion Criteria for testing at field laboratories
• Resting blood pressure >200 mmHg Systolic, and or >110 mmHg Diastolic
• Acute systemic infection (discuss with medical officer)
• AMS requiring treatment with acetazolamide, dexamethasone or nifedipine
• Acute chest pain
• New Arrhythmias or ECG changes
• Resting Arterial Oxygen Saturations <90% at sea level; <85% Kathmandu, <80% Namche <75% Pheriche, <70% at Everest Base Camp

**Criteria for stopping test**

• Excessive rise in blood pressure:
○ >250 mmHg Systolic; >115 mmHg Diastolic
• Drop in systolic blood pressure of >10 mmHg from baseline, with other indications of ischaemia (see below)
• >2 mm ST depression or >1 mm ST elevation
• Onset of angina or angina-like symptoms
• Onset of new arrhythmia other than ventricular ectopics
• Nervous system symptoms - ataxia, dizziness or near syncope
• Subject requests termination of test

**Figure 1:**
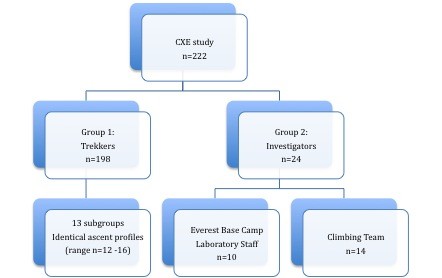
**Subject Groups in the Caudwell Xtreme Everest Study**.

Two groups of healthy volunteers were studied (figure 1). **Group 1 (trekkers) **were members of the public recruited by word of mouth and via public advertisement. **Group 2 (investigators) **were selected from the investigator team. The investigator team comprised 60 doctors, scientists, allied health professionals and medical students. Candidate investigators applied to CASE and were selected following interview (MG, KM). Criteria used to select group 2 subjects from the investigator team were previous event free exposure to high altitude (>4000 m) and previous experience of living in a harsh environment. Group 2 was divided into **base-camp laboratory staff **(who remained at Everest Base Camp for the duration of the expedition) and the **climbing team**. Criteria for participation in the climbing team were previous extreme altitude experience (event free ascents over 6500 m), general mountaineering experience (subjectively assessed by the expedition leader and climbing leader, MG and SD) and a demonstrated ability to conduct medical research at high altitude. An additional criterion for summit climbers was a previous successful event-free ascent over 8000 m.

### Setting

Baseline studies were performed at the investigators' human physiology laboratory at UCL (75 meters above sea level) between January 4th and February 26th 2007.

Field studies were completed between 31^st ^March and 6^th ^June 2007. Altitudes are expressed as meters above sea-level (m). Field studies were performed at dedicated laboratories set up by the investigator team at Kathmandu (1300 m), Namche Bazaar (3500 m), Pheriche (4250 m), Everest Base Camp (5300 m), Western Cwm (6400 m), South Col (7950 m) and the Balcony (8400 m). Laboratory altitudes, barometric pressures and inspired partial pressures of oxygen are summarised in table 2.

**Table 2:** Laboratory altitude, mean barometric pressure, mean laboratory temperature and inspired partial pressure of oxygen.

Laboratory	Approx Altitude metres	Ambient Temperature ˚C	Barometric Pressure millibar	Barometric Pressure mmHg	Barometric Pressure Kpa	**PiO**_**2 **_**mmHg**	**PiO**_**2 **_**Kpa**
**LONDON**	75	24.1 (1)	1005	754 (10)	100.5 (1.3)	148.0	19.7

**KATHMANDU**	1300	26.1 (1.5)	867	650 (3)	86.7 (0.4)	126.2	16.8

**NAMCHE**	3500	19.6 (2.6)	670	505 (3)	67.3 (0.4)	95.4	12.7

**PHERICHE**	4250	13.1 (1.7)	615	461 (2)	61.5 (0.3)	86.7	11.6

**EBC**	5300	21.5 (5.6)	538	404 (3)	53.8 (0.3)	74.7	9.9

**WCWM**	6400	12.7 (3.9)	467	350 (0.9)	46.7 (0.1)	63.4	8.5

**SOUTH COL**	7950	15.0 (8.9)	389	292 (2.3)	38.9 (0.3)	51.3	6.8

**BALCONY**	8400	Not recorded	363	272	36.3	47.1	6.3

Altitudes taken from map values to nearest 50 m; Barometric pressures and temperature are mean (standard deviation) values recorded during laboratory testing in the field; PiO2 = Inspired partial pressure of oxygen -calculated from barometric pressures.

### Intervention

**Figure 2:**
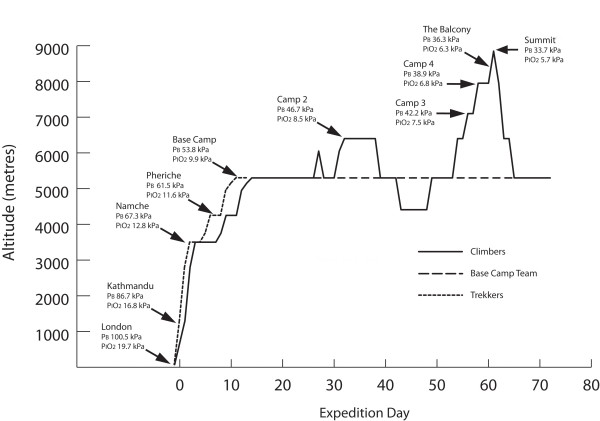
**Ascent profile, mean barometric pressure and mean PiO2 for Group 1 (Trekkers), Group 2: (Investigators: Climbers and Base Camp Team)**. Legend: Laboratories where testing was performed are labeled, intermediate altitudes indicate overnight stops without testing.

**Figure 3:**
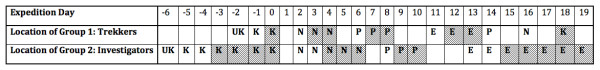
**Schedule of testing for Group 1 (Trekkers) and Group 2 (Investigators)**. Legend: Expedition day 1 was defined as the day of departure from Kathmandu. UK: United Kingdom; K: Kathmandu 1300 m; N: Namche 3500 m; P: Pheriche 4250 m; E: Everest Base Camp 5300 m. Shaded boxes: Testing days Unshaded boxes Group 1: arrival day at laboratory Unshaded boxes Group 2: arrival day at laboratory and/or laboratory set up day.

All subjects flew from London to Kathmandu (overnight). They then flew to Lukla (2800 m) in the Khumbu region and trekked to Everest Base Camp (EBC, 5300 m) (see figure 2). All subjects were sequentially tested at laboratories in Kathmandu, Namche Bazaar, Pheriche and Everest Base Camp. The time course and altitude ascent profiles for Group 1 and Group 2 are summarised in figures 2 and 3. Expedition day 1 was defined as the day of departure from Kathmandu. The ascent rate was chosen to minimise the incidence of high altitude illness and therefore maximise the number of subjects able to contribute data at the highest laboratory [16].

Group 1 (trekkers) was divided into 13 smaller groups of a maximum of 16 subjects. Two groups left the UK each week for the duration of the expedition. All Group 1 subjects followed an identical ascent profile arriving at EBC on day 11 (figures 2 and 3). All Group 2 (Investigator) subjects followed an identical ascent profile to EBC arriving on day 13. This differed from that of group 1 due to the logistical demands of establishing research laboratories and additional testing: additional time was thus spent in Kathmandu, and at Namche Bazaar, 3450 m (figure 3). On rest days during the ascent to EBC, excursions were strictly limited such that all subjects remained within 300 vertical metres of the laboratory altitude at all times in order to maintain an identical pattern of hypoxic exposure.

The laboratory staff, (n = 10), subsequently remained at EBC for the duration of the expedition (68 days). For these investigators, excursions were limited to within 500 vertical metres of the EBC altitude for the duration of the expedition. The climbing team (n = 14) followed an identical ascent profile until the completion of all testing at Camp 2 (Western Cwm), including identical acclimatisation outings (figure 2). The laboratory staff were not exposed to supplemental oxygen for the duration of the expedition. Climbers were not exposed to any supplemental oxygen until the completion of testing at Camp 2. All climbers used supplemental oxygen at flow rates of 2-4l/min for the summit climb above Camp 3 (7100 m) and at 0.5 l/min whilst sleeping at and above Camp 3. Testing was repeated at the end of the expedition (immediately prior to departure) for all group 2 subjects at EBC (days 66 to 71).

Group 1 subjects were consistently tested either on the day after arrival at any given altitude, or on the following day (Day 1 subjects, or Day 2 subjects: figure 3). For each subject the day of testing was kept constant to control for the effects of continued adaptation at the laboratory altitude. Furthermore, subjects were tested at the same time of day at all laboratories to control for diurnal variations in physiological responses. At Kathmandu, Namche and Everest Base Camp, group 2 subjects spent the first day after arrival setting up the lab; at Pheriche testing began on the first day after arrival. For group 2, testing was performed over 4 days at Kathmandu, 3 days at Namche, 2 days at Pheriche and 5 days at Everest Base Camp (figure 3). For the investigator group, the order of testing was kept constant for the core studies.

To minimise the confounding effects of hypoxic adaptation prior to the study period, all subjects refrained from any form of hypoxic training (hypoxic tents etc) and did not travel above 3000 m for 3 months prior to departure.

Subjects did not take prophylactic medication (eg acetazolamide) to prevent Acute Mountain Sickness (AMS). We developed and used a set of guidelines for the treatment of common altitude illnesses (eg AMS) and non-altitude conditions (eg acute upper respiratory tract infection, acute gastroenteritis), such that all individuals were treated in a standardized manner. All use of medications was recorded so that individuals receiving medication can be identified and where appropriate will be analyzed as a subgroup. For example, the use of acetazolamide in the treatment of AMS might have an effect on some of the resting physiological variables and we intend to undertake a subgroup analysis exploring this question.

### Measurements

#### Core studies (Groups 1 and 2)

All subjects from both groups were recruited into the 10 core studies unless exclusion criteria were present. The core studies were:

1. **Diary**: a daily diary of physiological variables and symptoms (see below).

2. **Maximum Exercise Capacity**: Incremental CPET using a standardized ramp protocol and breath-by-breath expired gas analysis was used (Metamax 3b, Cortex, Leipzig, Germany). Continuous vastus lateralis near infrared spectroscopy (NIRS) (InSpectraTM Tissue Spectrometer Model 325; Hutchinson Technology Inc, Hutchinson, MN, USA) [17] and continuous cerebral NIRS (Invos, Somanetics, MI, USA) were recorded at rest, throughout the incremental exercise protocol and during recovery. The lactate (or anaerobic) threshold was identified during incremental exercise testing as a change in the gradient of the VCO_2_-VO_2 _relationship (the V-slope method, [18]), typically accompanied by a systematic rise in the ventilatory equivalent for oxygen (VE/VO_2_) and in end tidal oxygen (P_ET_O_2_) without a concomitant decrease in end-tidal CO_2 _(P_ET_CO_2_) or increase in the ventilatory equivalent for CO_2 _(VE/VCO_2_) (ventilatory equivalents method) [19]. This method was successfully validated against direct arterial lactate measurements in a subgroup of subjects at altitude. The arterial lactate threshold was identified as the inflection point in the lactate response to incremental exercise above which there is a transition from a phase of no increase, or a small increase, to a phase of rapidly accelerating increase in blood lactate concentration [20].

3. **Metabolic Efficiency**: Exercise efficiency and exercise economy were evaluated using constant work rate cycling CPET (Metamax 3b, Cortex, Leipzig, Germany). Three steady state work rates below lactate threshold were assessed. Exercise economy was defined as the relationship between oxygen consumption and work rate during exercise; for exercise efficiency we calculated delta efficiency.

4. **Neurocognitive**: Test battery assessing cognition, attention, fine motor skills and memory: Trailmaking Test, Rey Test (auditory verbal learning), Letter Cancellation Test, Word Finding, Stroop Test, Block Design and Groove Peg Board test. A cohort of control subjects were tested using the same protocol and timings at sea-level in order to control for any learning effect in this study.

5. **Pupilometry**: The velocity and latency of the pupillary reflex response was assessed at each altitude using a hand-held ForSite Digital Pupillometer (Neuroptics Inc, Irvine, CA).

6. **Retinal Photography**: Assessing the distribution and depth of retinal haemorrhages using high-resolution digital photography: TopCon TRC NW200 (Tokyo, Japan).

7. **Spirometry**: Assessing standard spirometric indices including forced vital capacity, forced expiratory volume in one second and maximal voluntary ventilation using an ultrasonic flowmeter (New Diagnostic Designs, EasyOne^™^, NDD Medical Technologies, MA, USA)

8. **Systemic Oxygen Content**: Serial haemoglobin concentration (Hemocue AB, Hemocue, Sweden), haematocrit (microcentrifugation of whole blood using the Sigma 1-14 microcentrifuge, Sigma, Germany) and SpO_2 _(Nonin Onyx 9500, Nonin Medical Inc, Minnesota, USA)

9. **Plasma Biomarkers**: Serial inflammatory, metabolic and tissue injury plasma biomarkers and nitric oxide metabolites.

10. **Genes Associated with Hypoxia**

#### The Diary Study

All subjects in both study groups completed a physiological and symptom scoring diary for each day of the expedition using validated symptom scores. The Lake Louise symptom score [21], the Environmental Symptoms Questionnaire [22] and a novel headache score were recorded daily. The physiological profile included resting heart rate, blood pressure (BP), respiratory rate and arterial oxygen saturations (right index finger), repeated (with the exception of BP) after a standardised two minute step exercise challenge (CXE step test: 20 cm step, firm base, alternate feet sequentially, 1 step up or down per second by electronic metronome). Data collection for the diary study was completed shortly after waking each morning, prior to any oral intake. All subjects rested in a sitting position for a minimum of five minutes prior to recording resting measurements and subjects were blinded to their own measurements.

#### Additional Studies for Group 1 (Trekkers)

Subgroups of Group 1 were also included in smaller studies: muscle and cardiac magnetic resonance imaging (MRI) and 31P magnetic resonance spectroscopy (n = 7) [23], sleep at altitude (n = 15), smell (n = 59), hypoxic pulmonary vasoconstriction (n = 11) and performance of a functionally-closed oxygen delivery device (n = 6) (carried out after completion of all other testing).

#### Additional Studies for Group 2 (Investigators)

In addition to the core studies, all investigators participated in a weight and body composition study. Additional studies including olfactory and taste perception direct laryngoscopy, resting cerebral Doppler, ocular saccadometry and sub-lingual microcirculatory imaging [24] were performed on all Group 2 subjects. Subgroups of Group 2 were subjects for smaller studies: muscle biopsy (n = 19), gastric tonometry (n = 10), gastric emptying and nutrition (n = 12), lactate threshold CPET (n = 5), oxygen transport and arterial blood gas analysis (n = 10) [25], structural and volumetric 1.5T MRI (n = 21), functional cardiac and skeletal muscle nuclear magnetic resonance [23] (n = 7) and electrocardiogram (n = 17). Individuals for subgroup studies were selected on the basis of their planned maximum altitude and based on practical timetabling considerations.

### Study Design and Analysis Plan

The sample size of Group 1 reflected a compromise between the constraints imposed by the capacity of the available laboratories and the better predictive ability of a larger population to identify clinically relevant relationships between physiological variables and different conditions of environmental hypobaric hypoxia.

A central aim was to investigate changes in exercise efficiency and exercise economy in response to conditions of environmental hypobaric hypoxia. A second related aim was to explore the association between genetic variants and differences in physiological variables (eg exercise efficiency). A sample size of 198 gives a power of 0.88 for rejecting insignificant changes in exercise efficiency (based on mean delta efficiency of 25.8% with a standard deviation of 1.5% [26]) and 0.98 for rejecting insignificant single gene effects for an allele frequency of 10% with an alpha level of 5%. It is important to note that the number of subjects is of one order of magnitude greater than previous studies of this kind, with a consequent increase in statistical power.

Given the different ascent profiles and previous altitude exposure of the investigators, data arising from the two groups will be analysed separately and these analyses will broadly be performed in two stages. In the first stage, standard tests and regression models such as ANOVA and generalized linear models [27] will be employed for fitting changes in each observed variable as a function of relevant predictors. Three categories of such predictors are identified: base-line (sea-level) values for observed variables, covariation of other observed variables in hypoxia (at altitude) and genetic markers (presence or absence of specific alleles). Genetic markers (candidate genes) will be derived from promising candidates identified from transcriptomic and proteomic analyses of tissue samples obtained during this experiment in combination with pilot data from previous studies by our group and hypoxia responsive genes identified in the literature [13]. The aim of this first stage is to provide robust statistical data summaries describing the main trends in each variable during the ascent. Measures of predictive power obtained by cross-validation are a key component in guiding the refinement of the simple models used in this first phase.

In the second stage of the analysis we aim at enriching the data with plasma biomarker measurements obtained by laboratory analysis of samples collected during the expedition. Along with the simple models used for univariate analyses, these supplementary data represent the second key component for constructing a biological description of pathways responsible for hypoxia adaptation using multivariate hierarchical regression models [28]. The aim of this second stage of the analysis is to refine the description of the observed variables and to explore their relationships in light of the supplementary plasma biomarker data. Estimated conditional probabilities of variations in one variable as a function of the others measure the relative strength of the statistical relations leading to identifying the hypoxia adaptation pathway.

The key result of these statistical analyses in relation to the management of critical care patients is the ability to convert these estimated probabilistic relationships into early stage predictions of poor adaptation to hypoxia in critically ill patients using physiological, plasma and genetic biomarkers. These predictions can effectively act as a principled decision support system that may point to an opportunity to intervene with specific targeted therapies.

Statistical analyses will be performed mainly using standard and custom-made Matlab scripts (Mathworks, MA,USA) that will be made publicly available. SPSS (Mac v16 and Windows v15 SPSSinc Chicago, USA) will be used for preliminary analysis.

The main measures of goodness of fit and predictive power used will be p-values, posterior probabilities and cross-validation predictive probabilities for the observable variables.

## Results

Two hundred and eight volunteers applied to join the expedition as trekkers of whom four withdrew prior to baseline sea level testing for personal reasons. One volunteer was advised not to trek after medical screening because of severe respiratory disease requiring nocturnal non-invasive ventilation. Two hundred and three volunteers were tested at sea level and five of these withdrew prior to departure (one because of a back injury and four for personal reasons). In the light of findings at baseline exercise testing, six subjects were withdrawn from subsequent maximum CPET testing, and three of these were also withdrawn from the steady state CPET testing. Sixty-three applicants applied to join the investigator group of whom 60 were selected and able to participate. Twenty-four were selected to be investigator subjects (investigators) at EBC of whom 14 met criteria to become part of the climbing team (summit team = 10). One hundred and ninety eight trekkers (Group 1) and 24 investigators (Group 2) who had been tested in the UK commenced the trek. The baseline characteristics of the study groups are summarized in Table 3.

**Table 3:** Baseline Characteristics of the CXE study population

	Group 1 (Trekkers)	Group 2 (Investigators)	Group 2 Subgroup (Climbers)	Group 2 subgroup (Lab staff)
	**number (%)**	**number (%)**	**number (%)**	**number (%)**

**Total**	198 (100)	24 (100)	14 (100)	10 (100)

**Male**	125 (63)	18 (75)	12 (86)	6 (60)

**Previous Altitude Exposure (>3500 m)**	85 (43)	23 (96)	14 (100)	9 (90)

**Previous Extreme Altitude Exposure (>5000 m)**	37 (19)	21 (88)	14 (100)	7 (70)

**Smoker**	13 (7)	0 (0)	0 (0)	0 (0)

**Race - white**	191 (97)	22 (92)	12 (86)	10 (100)

	**Mean (SD)**	**Mean (SD)**	**Mean (SD)**	**Mean (SD)**

**Age**	44.7 (13.7) (range: 18-73)	35.2 (9.3) (range: 19-59)	36 (6.9) (range: 22-47)	34 (12.2) (range: 19-59)

**Height cm**	173 (9.2)	176 (7.0)	178.4 (5.9)	172.7 (2.2)

**Weight kg**	75.2 (13.6)	77.2 (12.4)	97.8 (0.9)	72.2 (9.5)

**BMI**	25.1 (3.2)	24.8 (2.9)	25.3 (3.3)	24.1 (2.2)

**Hb g/dl**	14.5 (1.2)	14 (0.9)	14.2 (0.7)	13.6 (0.9)

**Hct**	43.7 (2.6)	43.9 (3.4)	43.9 (2.1)	43.5 (3.2)

**SpO2%**	97.7 (1.6)	97.9 (1.0)	97.9 (1.1)	

**Oxygen content mls/l**	196.3 (16.2)	190.1 (11.6)	193.9 (10.0)	184.7 (12)

**VO2max mls**	2862 (799)	3624 (599)	3785 (505)	3398 (671)

**VO2max mls/kg**	38.2 (8.5)	47.2 (7.6)	47.4 (8.6)	47.0 (6.5)

Of 198 trekkers who left the UK, 190 (96%) reached Everest Base Camp. Eight did not arrive at EBC, due to acute mountain sickness in three subjects (1.5% of total) and non-altitude specific medical conditions in five (2.5% of total) (table 4). In the investigators group (group 2), all 24 subjects reached Everest Base Camp (table 5). Of the climbing team, all 14 subjects reached camp 2. Eight of ten summit climbers successfully reached the summit of Mount Everest. One member of the climbing team developed High Altitude Cerebral Oedema on arrival at Camp 3 (7100 metres, Day 56) during the final ascent to the summit. He was immediately treated with oxygen and dexamethasone and since it was after nightfall was stabilized overnight at Camp 3. At dawn he descended with assistance to Camp 2 and then EBC. He made a full recovery and did not subsequently ascend above EBC. One member of the climbing team turned back during the summit attempt with no altitude illness. One member of the laboratory staff was evacuated prior to the completion of the expedition from Pheriche (4250 metres, Day 54) with septic shock secondary to a severe community acquired pneumonia. The subject made a full recovery after treatment in Kathmandu. Subsequent investigations on return to the UK, revealed the subject had mild, previously undiagnosed bronchiectasis (table 5).

**Table 4:** Group 1 (Trekkers) - Subjects arriving at each laboratory and reasons for absence from laboratory

	Kathmandu (1300 m)	Namche (3500 m)	Pheriche (4250 m)	Everest Base Camp (5300 m)
No of Trekkers Arriving at Laboratory	198	197	195	190

Reason for Absence from Laboratory - gender, age, expedition day	nil absent	Respiratory Tract infection (1) *M, age 73, Day 1*	Abcess (1) *M, age 49, Day 3* AMS (1) *F, age 55, Day 3*	AMS (1) *F, Age 39, Day 10* Angina (1) *F, age 60, Day 7 * Respiratory Tract Infection (1) *M, age 68, Day 7* Diarrhoea and Vomiting (1) *F, age 59, Day 10* Recurrent Cluster Headache (1) *M, age 62, Day 8*

Notes: AMS = Acute Mountain Sickness; M = male; F = Female

**Table 5:** Group 2 (Investigators) - Number of subjects arriving at each laboratory and reasons for absence from the laboratory

	Kathmandu (1300 m)	Namche (3500 m)	Pheriche (4250 m)	EBC (5300 m)	WCwm (6400 m)	SCol (7950 m)	EBCend (5300 m)
Subjects at laboratory	24	24	24	24	14	12	23

Reason for absence from Laboratory - gender, age, expedition day	nil	nil	nil	nil	nil	HACE (1) *M,42, Day 56 * AMS (1) *F,35, Day 33*	Septic Shock (1) *M,59, Day 50*

HACE = high altitude cerebral oedema; AMS = acute mountain sickness; M = male; F = Female

The numbers of subjects tested in each protocol at each laboratory for group 1 and group 2 are summarized in tables 6 and 7 respectively. Mean laboratory pressures and laboratory temperatures are recorded in table 2.

**Table 6:** Group 1 (trekkers) (n = 198) - Testing performed at each laboratory

	Sea Level	Kathmandu (1300 m)	Namche (3500 m)	Pheriche (4250 m)	EBC (5300 m)
**Subjects at laboratory**	198	198	197	195	190

**CORE STUDIES**					

**Daily diary**	198	195	196	194	190

**CPX Ramp (+NIRS)**	190*	189	184	183	153

**CPX ME**	195**	n/a	191	n/a	164

**Spirometry**	198	197	190	176	185

**Venesection**	198	198	195	194	181

**Neurocognitive**	198	160 (on return)	195	n/a	185

**Pupillometry**	198	n/a	191	n/a	186

**Cranial measurements**	198	n/a	n/a	n/a	n/a

**Retinal photography**	183	n/a	n/a	n/a	183

**Plasma biomarkers**	198	198	195	194	181

**ADDITIONAL STUDIES**					

**Hypoxic Pulmonary Vasoconstriction**	31 screened 13 selected	n/a	11	n/a	11

**Smell and taste**	59	n/a	n/a	n/a	59

**Sleep**	n/a	n/a	n/a	n/a	15

**Oxygen Delivery Circuit**	n/a	n/a	n/a	n/a	6

**Cardiac MRI and 31P-MRS**	7	n/a	n/a	n/a	n/a

**Skeletal Muscle MRI and 31P-MRS**[23]	7	n/a	n/a	n/a	n/a

**Structural and Volumetric MRI Study**	7	n/a	n/a	n/a	n/a

* 6 subjects withdrawn from incremental CPET prior to departure and 2 because of poor baseline data quality

** 3 subjects withdrawn from efficiency CPET prior to departure

**Table 7:** Group 2 (Investigators) (n = 24) - Testing performed at each Laboratory

	Archway (75 m)	Kathmandu (1300 m)	Namche (3500 m)	Pheriche (4280 m)	EBC (I) (5300 m)	EBC (II) (5300 m)	Camp 2 (6400 m)	South Col (7950 m)
**Subjects at laboratory**	24	24	24	24	24	23	14	12

**CORE STUDIES**								

**Daily diary**	24	24	24	24	24	23	14	5

**CPX Ramp (+muscle and brain NIRS)**[17]	24	24	22	24	23	22	14	5

**CPX ME**	24	24	23	n/a	24	23	14	n/a

**Spirometry**	24	24	24	23	23	23	14	6

**Venesection**	24	24	24	24	24	23	14	n/a

**Neurocognitive**	21	21 (on return)	21	21	21	n/a	13	6

**Pupillometry**	24	24	24	20	24	23	14	0

**Cranial measurement**	24	n/a	n/a	n/a	n/a	n/a	n/a	n/a

**Retinal Photography**	24	n/a	n/a	n/a	24	23	n/a	n/a

**Plasma biomarkers**	24	24	24	24	24	23	n/a	n/a

**ADDITIONAL STUDIES**								

**Arterial Blood Gases**[25]	10	n/a	n/a	n/a	9	n/a	9	n/a

**Gastric Tonometry**	10	n/a	n/a	n/a	n/a	9	n/a	n/a

**Cardiac Output**	10	n/a	n/a	n/a	n/a	9	n/a	n/a

**Microcirculation**[24]	24	n/a	24	n/a	24	23	14	4

**Weight & Body comp**	24	24	24	24	24	23	13	n/a

**Muscle Biopsy**	20	n/a	n/a	n/a	n/a	18	n/a	n/a

**ECG**	19	19	19	19	19	19	n/a	n/a

**Oxygen circuit**	n/a	n/a	n/a	n/a	n/a	n/a	5	n/a

**Smell and taste**	24	n/a	n/a	n/a	n/a	24	n/a	n/a

**Thromboelastography**	17	n/a	n/a	17	n/a	14	n/a	n/a

**Cerebral Doppler**	24	24	24	24	24	24	13	5

**Saccadometry**	22	22	22	22	22	22	12	n/a

**Cardiac MRI and 31P-MRS**	2	n/a	n/a	n/a	n/a	n/a	n/a	n/a

**Skeletal Muscle MRI and 31P-MRS**	7	n/a	n/a	n/a	n/a	n/a	n/a	n/a

**MR Brain structural and volumetric studies**	15	n/a	n/a	n/a	n/a	n/a	n/a	n/a

Published manuscripts reporting data from CXE to date are:

1) Microcirculatory changes at altitude in the investigator group (n = 24) [24]

2) Muscle NIRS responses at altitude in the investigator group (n = 24) [17]

3) Arterial blood gases and oxygen content in the investigator group (n = 10) [25]

4) Skeletal muscle energetics (P-MRS) in 7 trekkers and 7 climbers [23].

## Discussion

### Statement of principal findings

We have demonstrated the feasibility of safely conducting a large (>200 subject) study at extreme altitude with a consistent pattern of hypoxic exposure. The study was designed to explore inter-individual variability in adaptation to hypoxia rather than the determinants of altitude related illness. To this end, the slow ascent rate was planned to avoid a significant incidence of altitude related illness. The effectiveness of this strategy was reflected in the high rate of success in reaching Everest Base Camp when compared with previous reports of ascents in this area and to the low incidence of missed tests [16]. Consequently the completion rate for planned testing was more than 99% in the investigator group and more than 95% in the trekkers overall. All equipment was tested for reliability and validity in hypobaric and environmental chambers (-25 degrees Celsius) and most devices were tested in the field during three pilot expeditions to the Alps (2006) and the Himalaya (2005, 2006) prior to the CXE expedition. The very low rate of equipment failure was an additional factor in the high rate of successfully completed tests. In particular, validation of the cardiopulmonary exercise testing equipment was an important element of expedition preparation, as breath-by-breath equipment had not previously been validated at extreme altitude. As a result of this preparation, only one exercise test was not completed for technical reasons. The pilot expeditions also permitted investigators to gain practical experience of the experimental protocols in field conditions and modify them where necessary.

### Strengths and weaknesses of the study

Strengths of this study are the large number of subjects (for a study in this environment), the matched ascent profile, sea-level control data, and the high rate of test completion. Large subject numbers provide the statistical power to discriminate between, and identify associations with, different patterns of adaptation as well as to detect differences in phenotypic response by somatic genotype (prospective gene-environment interaction study). Matched subject ascent profiles and baseline measurements at sea-level control for variability of exposure to hypoxia and thereby permit valid inter-individual comparison of responses to hypoxia (with subjects as their own controls), maximizing the signal (true physiological differences) to noise (variations in exposure to environmental hypoxia) ratio. In the neurocognitive study, where a learning effect was a significant risk, we recruited a parallel control group who were studied over the same time-scale as the altitude exposed subjects, but remained at sea-level. Fifty seven percent of the trekker cohort (Group 1) were altitude naïve. This sub-group is therefore not confounded by self-selection due to prior altitude tolerance.

Weaknesses of this study include potential bias resulting from the method of recruitment and selection of the subjects (non-random sample). However, randomisation to environmental hypoxia exposure was not considered a feasible option for this type of study (opportunistic observation of individuals with a desire to visit the study environment). Several factors suggest that the trekker group is not representative of a 'normal' population (eg gender distribution; prevalence of previous altitude exposure, exercise capacity). In order to explore this, we will undertake subgroup analyses investigating the influence of these factors on our findings. In the investigator group self-selection due to previous altitude exposure is a likely source of bias. In particular, the summit team climbers had all experienced an event-free ascent over 8000 m. Investigator and trekker groups will therefore be analysed separately.

Uncertain validity of measurements at high altitude is another potential weakness that was minimised by prior testing and validation of measurement devices in cold and hypobaric chamber facilities and in the field. Environmental factors such as ambient temperature, subject dehydration and concurrent illnesses may also have confounded results. However laboratory temperature was much less variable than ambient temperature: for example, mean laboratory temperature at Everest Base Camp was 21.5 ˚C and the minimum and maximum temperatures recorded in laboratories during testing (including the South Col) were 4.6˚C and 33˚C respectively (table 2). All subjects were encouraged to maintain good hydration (guided by the production of good quantities of pale urine) and plasma osmolality remained constant. A detailed reporting system for medical problems was used to identify those subjects in whom intercurrent illness was a potential confounder. The similarity between data derived from chamber (eg Operation Everest II [29]) and field studies (eg AMREE [8,30]) implies that hypoxia is the over-riding physiological stimulus in field studies and argues against substantial confounding by environmental factors.

The use of supplemental oxygen at and above 7100 metres is also a potential weakness of this study. Supplemental oxygen was used for safety reasons; deaths above 8000 metres occur twice as frequently in individuals breathing ambient air than in those using supplemental oxygen [31]. To minimise the effect of this on our observations, all measurements were made after at least 20 minutes breathing ambient air at which time any additional oxygen would have been "washed out" by the high levels of ventilation that occur at such altitudes [25]. However, intermittent exposure to low flow (0.5-2 litres) oxygen supplementation may have altered the trajectory of adaptation in these subjects and we cannot know whether our observations would have differed had they been made in climbers who had never used supplemental oxygen. The effect of supplemental oxygen on arterial oxygen saturations is dependent on oxygen flow rate, delivery system and minute ventilation and varies between individuals [32,33]. Importantly, this was not an issue for the trekker cohort (n = 198) as subjects were not exposed to supplemental oxygen prior to measurements being made.

We avoided the use of interventions to test candidate mechanisms and the descriptive nature of the data may be considered a weakness of this study. However, the variety of outputs from different measurement techniques (e.g. genomics, proteomics, plasma biomarkers and functional MRI) allows observation of consistent patterns of response that may be strongly suggestive of particular mechanisms.

The generalisability/external validity of data from this type of study with respect to pathophysiological conditions in critical illness and other "hypoxic" conditions rests on the validity of the underlying model, which remains uncertain. Accepted approaches to the study of hypoxic adaptation in critical illness have included cellular (in vitro and ex vivo) [34], animal (in vivo) [35] and computer (in silico) models [36]. In vivo studies are considered to provide the most valid models of human critical illness because they utilise integrative mammalian physiology. However, discordance between animal and human studies has raised concerns about the limitations of these models [37,38]. Specifically there is concern that in vivo models, fail to match the complexity of human physiology in the acutely ill patient [39]. This led us to propose that the study of healthy humans, progressively exposed to environmental hypobaric hypoxia in a controlled manner, during an ascent to high altitude, may be a valid model for exploring adaptation to hypoxia in the critically ill: [1,5].

Furthermore, there is supportive evidence in the form of common genetic determinants of performance at altitude and outcome in hypoxic critical illness [40,41]. The I allele of the insertion/deletion Angiotensin Converting Enzyme (ACE) polymorphism is over-represented in climbers who have successfully ascended over 7000 metres (without supplemental oxygen)[41] and 8000 metres [42]. The same I allele is associated with increases survival in the Adult Respiratory Distresss Syndrome (ARDS), an archetypal hypoxic illness, in critically ill patients[40,43].

### Strengths and weaknesses in relation to other studies

In comparison to previous field and laboratory studies of adaptation to hypoxia in humans, our study is unique in both scale and in the variety of measurements made in a cohort of subjects with matched hypoxic exposure. Several chamber experiments (Operation Everest 1, 2 and 3) have studied small cohorts (n = 4-8) of subjects in considerable detail, but the small sample sizes have prevented meaningful exploration of inter-individual differences [29,44,45]. Previous comprehensive altitude studies have studied smaller numbers of subjects with un-matched ascent profiles [6-8]. Our study provides a unique opportunity to explore inter-individual differences.

We explored the possibility of conducting this study in a hypobaric chamber, but chose a field study for the following reasons. Recruiting subjects for a three-week chamber study (in comparison with a trek to EBC) would have been considerably more difficult. In addition this approach might have incurred significant additional costs; our subjects were entirely self-funded (and contributed towards the costs of the research) whereas volunteers in chamber studies often expect remuneration. Furthermore, the high cost of running a chamber study (continuous medical and technical cover) and limited availability of long duration hypoxia facilities capable of accommodating large cohorts contributed to this decision.

### Unanswered questions and future research

Further research arising from this study will follow two themes. First, studies in patients to explore the validity of the model by applying the findings of this study to pathophysiological problems in clinical settings [46,47]. Second, collecting additional healthy volunteer data from subjects exposed to hypoxia in further field studies and chamber studies. Future studies using this model of field study might answer additional questions by using alternative or additional measurement techniques, or trialing novel interventions. Studying highland residents and comparing patterns of physiological response with lowland visitors to altitude, and studying responses in younger subjects, amongst whom data is very limited would also be valuable.

## Conclusions

This study demonstrates the feasibility and safety of conducting a large healthy volunteer cohort study of human adaptation to hypoxia in this difficult environment. Systematic measurements of a large set of variables were achieved with high fidelity in 222 subjects and at altitudes of up to 5300 metres. Hypoxic exposure was successfully standardized allowing interrogation of inter-individual variability in hypoxic adaptation. The resulting dataset is a unique resource for the study of genotype:phenotype interactions in relation to hypoxic adaptation which may improve our understanding of responses to hypoxia in critical illness.

## List of Abbreviations used

EBC: Everest Base Camp; CASE: Centre for Altitude, Space and Extreme Environment Medicine, UCL; CXE: Caudwell Xtreme Everest; EHH: Environmental Hypobaric Hypoxia

## Competing interests

The authors declare that they have no competing interests.

## Authors' contributions

DL contributed to the conception and design of the study, to the acquisition of data, the analysis of data, the interpretation of data and drafted the manuscript. DM contributed to the conception and design of the study, to the acquisition of data, the analysis of data, the interpretation of data and helped to draft the manuscript. His contribution to the manuscript was equal to DL's. MW contributed to the conception and design of the study, to the acquisition of data, the analysis of data, the interpretation of data and revision and approval of the manuscript. KM contributed to the conception and design of the study, to the acquisition of data, the analysis of data and to the revision and approval of the manuscript. SD contributed to the conception and design of the study, to the acquisition of data, and to the revision and approval of the manuscript. MM contributed to the conception and design of the study, to the acquisition of data, the interpretation of data and revision and approval of the manuscript. FR contributed to the analysis and interpretation of data and helped to draft the manuscript. HM contributed to the conception and design of the study, to the interpretation of data and to the drafting of the manuscript. MG contributed to the conception and design of the study, to the acquisition of data, the analysis of data, the interpretation of data and helped to draft the manuscript.

All authors read and approved the final draft.

## Pre-publication history

The pre-publication history for this paper can be accessed here:

http://www.biomedcentral.com/1471-2288/10/98/prepub
